# Ankyrin Repeat Domain 1 is Up-regulated During Hepatitis C Virus Infection and Regulates Hepatitis C Virus Entry

**DOI:** 10.1038/srep20819

**Published:** 2016-02-10

**Authors:** Thoa T. Than, Giao V. Q. Tran, Kidong Son, Eun-Mee Park, Seungtaek Kim, Yun-Sook Lim, Soon B. Hwang

**Affiliations:** 1 National Research Laboratory of Hepatitis C Virus, Ilsong Institute of Life Science, Hallym University, Anyang, South Korea; 2 Institute of Gastroenterology, Department of Internal Medicine, Yonsei University College of Medicine, Seoul, South Korea

## Abstract

Hepatitis C virus (HCV) is highly dependent on host proteins for its own propagation. By transcriptome sequencing (RNA-Seq) analysis, we identified 30 host genes that were significantly differentially expressed in cell culture-grown HCV (HCVcc)-infected cells. Of these candidate genes, we selected and characterized ankyrin repeat domain 1 (ANKRD1). Here, we showed that protein expression of ANKRD1 was up-regulated in HCVcc-infected cells. We further showed that protein expression level of ANKRD1 was increased by nonstructural 5A (NS5A) protein. ANKRD1 specifically interacted with NS5A both *in vitro* and coimmunoprecipitation assays. Protein interaction was mediated through the domain II of NS5A and the C-terminal region of ANKRD1. Promoter activity of ANKRD1 was also increased by NS5A protein. Moreover, up-regulation of ANKRD1 expression was mediated through alteration in intracellular calcium homeostasis and ER stress in HCVcc-infected cells. We showed that silencing of ANKRD1 impaired HCV propagation without affecting HCV replication. By using HCV-like infectious particle (HCV-LP), we demonstrated that HCV single-cycle infection was drastically impaired in ANKRD1 knockdown cells. Finally, we verified that ANKRD1 was required for HCV entry. These data suggest that HCV coopts ANKRD1 for its own propagation and up-regulation of ANKRD1 may contribute to HCV-mediated liver pathogenesis.

Hepatitis C virus (HCV) infection causes chronic liver diseases, including steatosis, cirrhosis, and hepatocellular carcinoma^1^,^2^,^3^,^4^. An estimated 170 million individuals are chronically infected with HCV worldwide and more than 350,000 people die each year from HCV-associated liver diseases^4^,^5^. HCV is a small enveloped virus with a positive-sense, single-stranded RNA genome that encodes a large polyprotein of 3010 amino acids. This polyprotein is processed by viral and cellular proteases to yield structural (core, E1, and E2) and nonstructural (p7, NS2, NS3, NS4A, NS4B, NS5A, and NS5B) proteins^6^. Until recently, standard therapy for HCV patient is the combination of pegylated interferon-α and ribavirin. However, this therapy shows a sustained virologic response with significant differences among genotypes and patients’ situations. Recently, U.S. FDA approved several direct-acting antivirals (DAAs). However, sky-high price of these new drugs makes unaffordable for majority of HCV infected patients^7^. Moreover, there are still potential occurrences of resistant variants due to inherent characteristics of RNA virus. Since HCV requires host cellular proteins for its own replication, targeting host proteins will be an alternative strategy to overcome the low genetic barrier to resistance. By transcriptome sequencing (RNA-Seq) analysis, we identified 30 host genes that were highly differentially expressed in cell culture-grown HCV (HCVcc)-infected cells. Among these, ankyrin repeat domain 1 (ANKRD1) was selected for further characterization.

ANKRD1, also known as cardiac ankyrin repeat protein (CARP), is a pleiotropic functional protein belong to a conserved family of muscle ankyrin repeat protiens^8^,^9^. ANKRD1 is discovered as a novel cytokine-inducible nuclear protein in endothelial cells^10^,^11^. ANKRD1 contains a nuclear localization signal, a PEST-like sequence, four repeats of an ankyrin motif, and multiple phosphorylation consensus sites^10^. ANKRD1 acts as a transcriptional regulator in cardiomyogenesis^12^. ANKRD1 is expressed at the highest levels in skeletal muscle and heart where they are localized to the I band of the sarcomere through binding to titin and myopalladin^12^. ANKRD1 interacts with many proteins, including cardiac calcium-handling protein calsequestrin-2 (CASQ2)^13^, the Y-box transcription factor 1 (YB-1)^14^, the intermediate filament protein desmin, and the muscle-specific RING finger ubiquitin ligases MuRF1/MuRF2^15^. It has been reported previously that ectopic expression of ANKRD1 led to enhanced apoptotic cell death in hepatoma cells^16^. Although ANKRD1/CARP protein has been intensively studied, its role in liver disease remains largely unknown.

In the present study, we demonstrated that protein expression of ANKRD1 was up-regulated in HCVcc-infected cells. We further showed that ANKRD1 expression level was increased by NS5A. Furthermore, ANKRD1 was specifically interacted with NS5A both *in vitro* and coimmunoprecipitation assays. Promoter activity of ANKRD1 was also increased by NS5A protein. In addition, the up-regulation of ANKRD1 was mediated through ER stress. By single-cycle infection assay using HCV-like infectious particle (HCV-LP), we showed that HCV propagation was drastically impaired in ANKRD1 knockdown cells. Finally, we demonstrated that ANKRD1 was involved in the entry step but not binding step during HCV infection. These data suggest that ANKRD1 may represent a novel entry factor required for HCV infection.

## Results

### HCV up-regulates ANKRD1 expression

**Figure 1:**
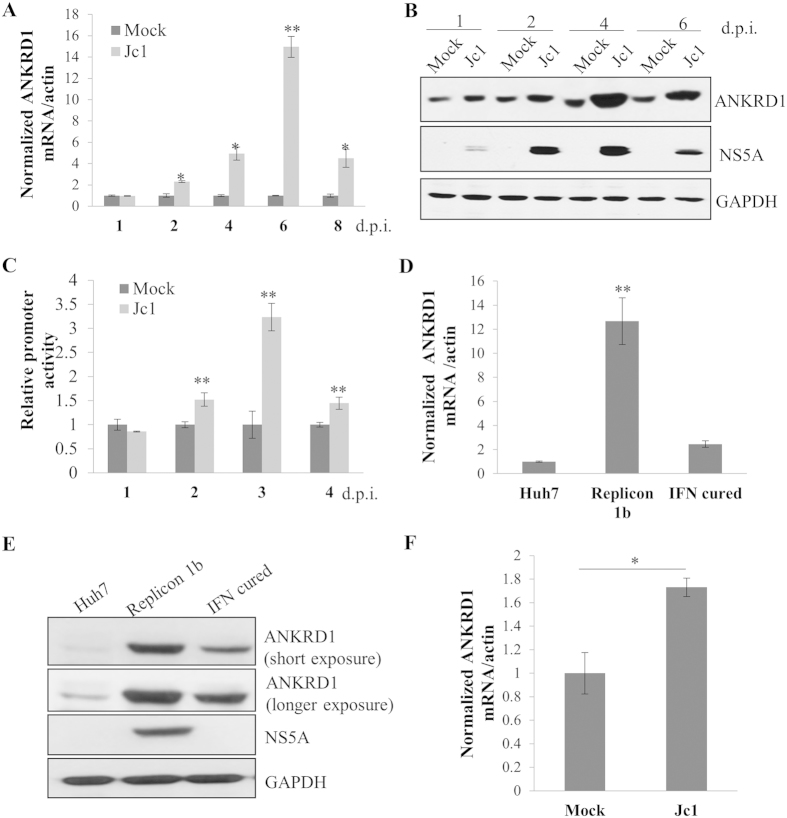
HCV up-regulates ANKRD1 expression level. (**A**) Huh7.5 cells were either mock infected or infected with HCV Jc1 for 4 h and then mRNA levels of ANKRD1 were analyzed by qRT-PCR at the indicated time points. d.p.i., days postinfection. (**B**) Total cell lysates were immunoblotted with the indicated antibodies at the indicated time points. (**C**) Huh7.5 cells were either mock infected or infected with Jc1 for 4 h. Cells were further transfected with ANKRD1-luc prompter reporter plasmid at one day prior to the indicated d.p.i. Cells were harvested and luciferase activities were determined at the indicated time points. Data from two independent experiments were quantified. (**D**) mRNA levels of ANKRD1 in Huh7 cells, IFN cured cells, and replicon cells derived from HCV genotype 1b were determined by qRT-PCR. (**E**) Protein levels of ANKRD1 in Huh7 cells, IFN cured cells, and replicon cells were immunoblotted with the indicated antibodies. (**F**) Human primary hepatocytes were infected with Jc1 for 4 h and harvested at 4 days postinfection. Total RNAs were extracted and ANKRD1 mRNA levels were quantified by qRT-PCR. Data represent averages from at least three independent experiments for panels (**A**,**C**,**D**,**F**). The asterisks indicate significant differences (*P < 0.05; **P < 0.01) from the value for the control.

By employing RNA-seq technology, we observed that 145 genes were up-regulated more than two-fold during Jc1 infection as compared with mock infection (Supplementary Table 1). By performing qRT-PCR analysis, we further verified that 30 genes were highly differentially expressed in HCVcc-infected cells (Supplementary Fig. S1). Of these, we selected ANKRD1 for further characterization because this gene displayed the highest effect on HCV propagation (Supplementary Fig. S2). Figure 1A shows that ANKRD1 mRNA level of ANKRD1 was dramatically increased in HCV infected cells as compared with mock infected cells. Consistently, protein level of ANKRD1 was elevated during the course of HCV infection (Fig. 1B). To investigate if transcriptional level of ANKRD1 was also regulated by HCV infection, Huh7.5 cells were transfected with luc reporter plasmid consisting of −2000 nt to ± 25 nt of ANKRD1 promoter and then infected with Jc1. Figure 1C shows that ANKRD1 promoter activities were gradually increased during the course of HCV infection. In addition, mRNA level of ANKRD1 was also significantly increased in replicon cells as compared with Huh7 and IFN cured cells. We further verified that protein level of ANKRD1 was also remarkably augmented in replicon cells (Fig. 1E). These results indicate that ANKRD1 gene expression may be up-regulated by HCV nonstructural protein. Finally, we verified that ANKRD1 mRNA level of ANKRD1 was significantly increased in HCV infected human primary hepatocytes (Fig. 1F). Together, these data indicate that HCV positively regulates ANKRD1 expression.

### ANKRD1 expression is up-regulated by HCV NS5A

**Figure 2:**
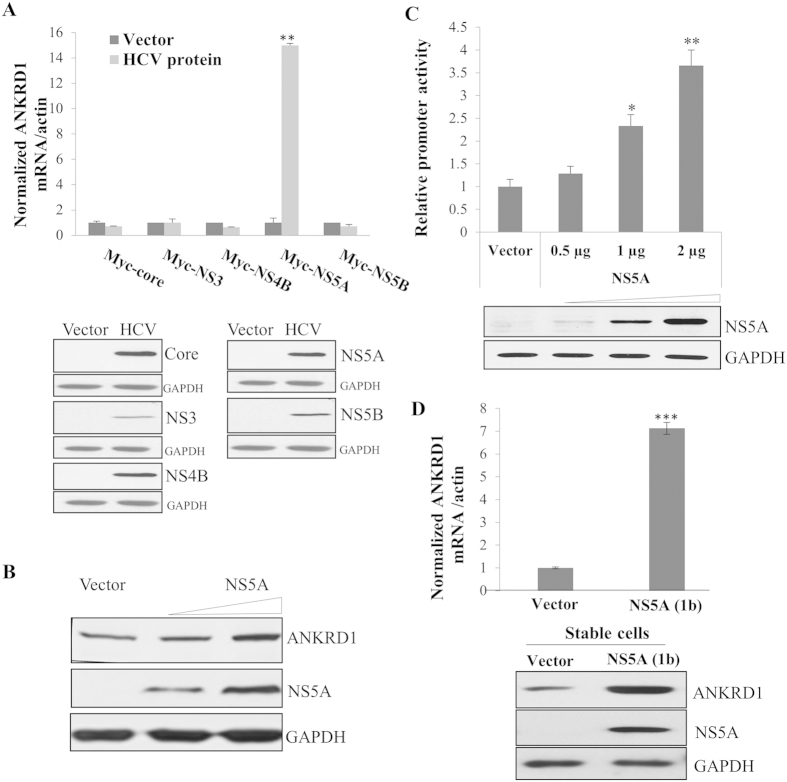
ANKRD1 expression level is upregulated by NS5A protein. (**A**) (Upper) Huh7.5 cells were transiently transfected with either vector or the indicated HCV protein expression plasmid, individually. At 48 h after transfection, ANKRD1 mRNA levels were analyzed by qRT-PCR. Data represent averages from at least three independent experiments. The asterisks indicate significant differences (**P < 0.01) from the value for the control. (Lower) HCV protein expressions were verified by immunoblot analysis using the indicated antibodies. (**B**) Huh7.5 cells were transiently transfected with either vector or increasing amounts of Myc-tagged NS5A expression plasmid. At 5 days after transfection, total cell lysates were immunoblotted with the indicated antibodies. (**C**) Huh7.5 cells were transfected with ANKRD1-Luc reporter plasmid with either vector or increasing amounts of Myc-tagged NS5A expression plasmid. At 48 h after transfection, cells were harvested and luciferase activities were determined. Data represent averages from at least three independent experiments. The asterisks indicate significant differences (*P < 0.05; **P < 0.01) from the activity for the vector control. (**D**) (Upper panel) mRNA levels of ANKRD1 in either vector stable or NS5A stable cells derived from genotype 1b were quantitated by qRT-PCR. Asterisks indicate significant difference (***P < 0.001). (Bottom panel) Total cell lysates harvested from either vector stable or NS5A stable cells were immunoblotted with the indicated antibodies.

Since ANKRD1 expression level was increased in both HCV replicon cells and HCV infected cells, we investigated if any of the HCV protein was responsible for this regulation. Huh7.5 cells were transiently transfected with either vector or Myc-tagged core, NS3, NS4B, NS5A, NS5B, individually for 48 h and then mRNA levels of ANKRD1 were analyzed by qRT-PCR. As shown in Fig. 2A, ANKRD1 mRNA level was significantly increased by NS5A but not by other HCV proteins. To verify this result, Huh7.5 cells were transfected with increasing amounts of plasmid expressing NS5A protein. Figure 2B shows that ANKRD1 protein level was gradually increased by NS5A. Moreover, promoter activities of ANKRD1 were also augmented by NS5A in a dose dependent manner (Fig. 2C). To corroborate these results, we quantified both mRNA and protein levels of ANKRD1 in NS5A stable cells. Figure 2D shows that both mRNA and protein levels of ANKRD1 were significantly augmented in NS5A stable cells as compared with vector stable cells. Collectively, these data clearly indicate that NS5A plays a crucial role in modulating ANKRD1 gene expression in HCV replicating cells.

### ANKRD1 interacts with NS5A through domain II of NS5A and C-terminal half of ANKRD1

**Figure 3:**
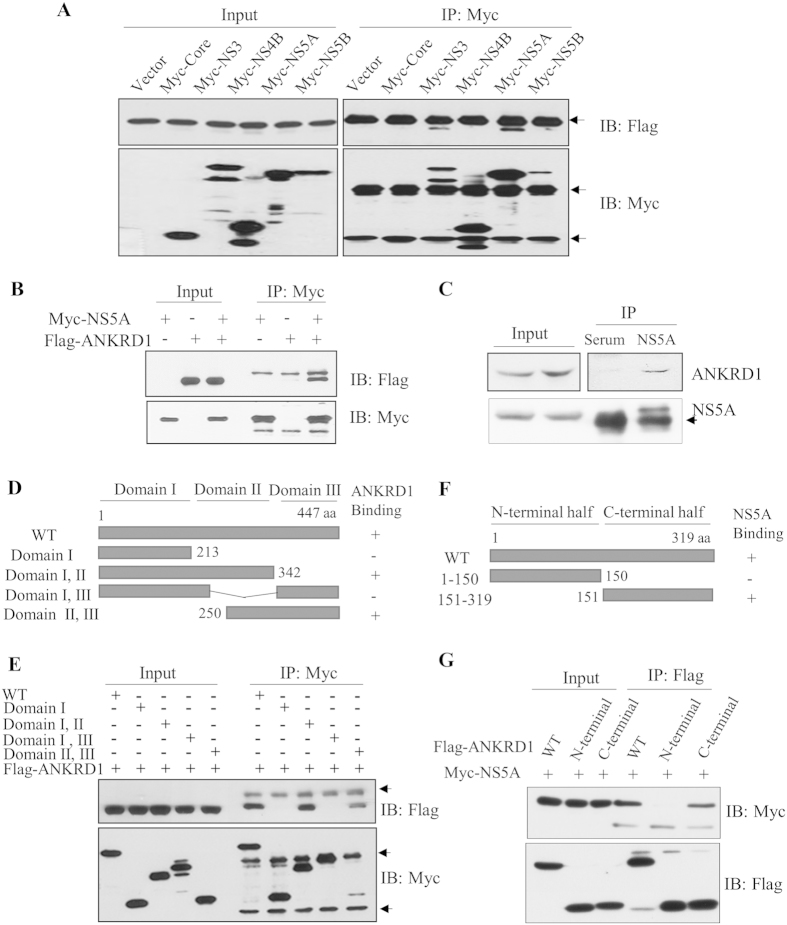
ANKRD1 interacts with NS5A protein through domain II of NS5A and C-terminal half of ANKRD1. (**A**) HEK293T cells were cotransfected with Flag-tagged ANKRD1 and vector or the indicated each of Myc-tagged HCV protein expression plasmid, respectively. At 48 h after transfection, cell lysates were immunoprecipitated with anti-Myc antibody and then bound proteins were detected by immunoblot analysis using anti-Flag antibody. Arrows indicate IgG. (**B**) HEK293T cells were cotransfected with Myc-tagged NS5A and Flag-tagged ANKRD1. At 48 h after transfection, cell lysates were immunoprecipitated with an anti-Myc antibody, and bound proteins were detected by immunoblotting with an anti-Flag antibody. (**C**) Huh7.5 cells were electroporated with 10 μg of Jc1 RNA. Cell lysates harvested at 4 days after electroporation were immunoprecipitated with either control IgG or an anti-NS5A antibody. Bound protein was detected using an anti-ANKRD1 antibody (upper panel). Immunoprecipitation efficiency was verified using an anti-NS5A antibody (lower panel). (**D**) Schematic diagram of both wild-type and mutant constructs of NS5A. (**E**) HEK293T cells were cotransfected with Flag-tagged ANKRD1 and various constructs of Myc-tagged NS5A expression plasmid. At 48 h after transfection, cell lysates were immunoprecipitated with an anti-Myc monoclonal antibody and then bound proteins were immunoblotted with an anti-Flag antibody. The arrowheads denote IgG. (**F**) Schematic diagram of both wild-type and mutant constructs of ANKRD1. (**G**) HEK293T cells were cotransfected with Myc-tagged NS5A and various constructs of Flag-tagged ANKRD1 expression plasmid. At 48 h after transfection, cell lysates were immunoprecipitated with an anti-Flag monoclonal antibody and bound proteins were immunoblotted with an anti-Myc antibody.

Since ANKRD1 expression was up-regulated in both HCV replicon cells and HCV infected cells, we speculated that ANKRD1 might interact with viral proteins. For this purpose, HEK293T cells were cotransfected with Flag-tagged ANKRD1 and each of Myc-tagged viral protein expression plasmid. Protein interaction was determined by coimmunoprecipitation assays. Figure 3A shows that ANKRD1 interacted with both HCV NS3 and NS5A proteins. Since ANKRD1 expression was up-regulated only by NS5A and NS5A interacted with ANKRD1 stronger than NS3, we further characterized protein interaction between NS5A and ANKRD1. As shown in Fig. 3B, ANKRD1 specifically interacted with NS5A protein. To verify this interaction, Huh7.5 cells were electroporated with 10 μg of Jc1 RNA. Cell lysates harvested at 4 days after electroporation were immunoprecipitated with rabbit anti-NS5A antibody. Bound protein was detected by immunoblot analysis using an anti-ANKRD1 antibody. Indeed, endogenous ANKRD1 interacted with NS5A protein in HCV replicating cells (Fig. 3C). We further verified that ANKRD1 interacted with NS5A in GST pulldown assay (Supplementary Fig. S3). To determine the region in NS5A responsible for ANKRD1 binding, the interactions between ANKRD1 and various deletion mutants of NS5A (Fig. 3D) were determined by a transfection-based coimmunoprecipitation assay. As shown in Fig. 3E, ANKRD1 interacted with mutants encompassing domain I, II and II, III but not with other mutants of NS5A. This result indicated that domain II of NS5A was responsible for binding with ANKRD1. Next, we determined the region in ANKRD1 for NS5A binding. We constructed both N-terminal- and C-terminal-truncated mutants (Fig. 3F), and the binding domain was determined as described above. Figure 3G showed that NS5A interacted with a mutant harboring the C-terminal half of the ANKRD1 but not with a mutant harboring the N-terminal half of the ANKRD1, indicating that NS5A interacted with ANKRD1 through the region encompassing the 4 tandem ankyrin-like repeats (ANK) in the C-terminal region.

### HCV up-regulates ANKRD1 expression via ER stress

**Figure 4:**
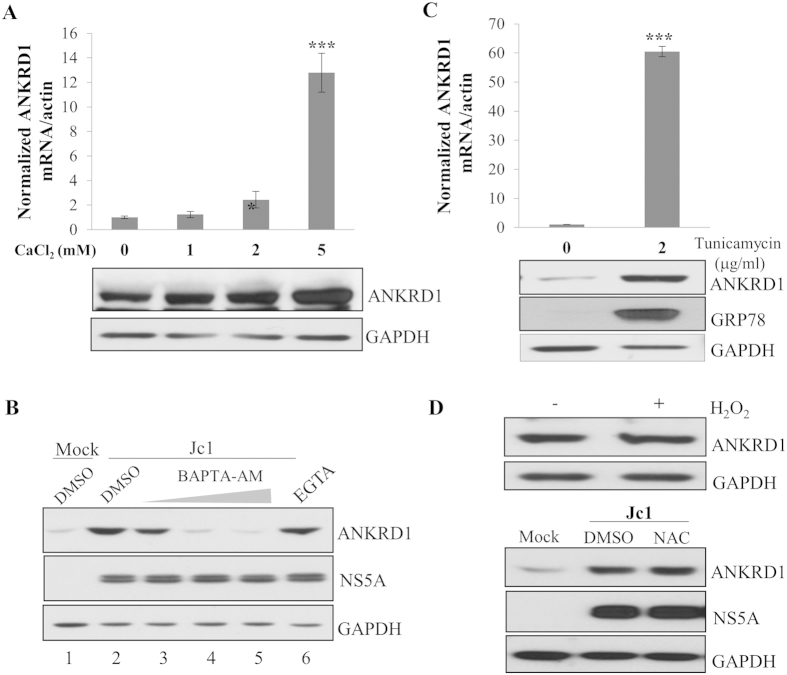
HCV up-regulates ANKRD1 expression via ER stress. (**A**) Huh7.5 cells were either left untreated or treated with various doses of CaCl_2_ for 16 h. Both mRNA and protein levels of ANKRD1 were analyzed by qRT-PCR (upper) and immunoblot analysis (lower), respectively. (**B**) Huh7.5 cells were either mock infected or infected with Jc1 for 6 days. The cells were either left untreated or treated with various doses (5, 10, 20 μM) of intracellular Ca^2+^ chelator, BAPTA/AM. Twenty μM of EGTA, extracellular Ca^2+^ chelator, was used as a control. Total cell lysates were immunoblotted with the indicated antibodies. (**C**) Huh7.5 cells were either left untreated or treated with 2 μg/ml of tunicamycin. Both mRNA and protein levels of ANKRD1 were analyzed by qRT-PCR (upper) and immunoblot analysis (lower), respectively. GRP78 was detected as a ER stress marker. (**D**) (Upper panel) Huh7.5 cells were either left untreated or treated with 500 μM of H_2_O_2_ for 24 h. Total cell lysates were immunoblotted with the indicated antibodies. (Lower panel) Huh7.5 cells were either mock infected or infected with Jc1. At day 3 postinfection, cells were either left untreated or treated with 20 μM of NAC for 24 h. Total cell lysates were immunoblotted with the indicated antibodies.

It has been reported previously that HCV regulates Ca^2+^ and ROS signaling cascade^17^,^18^,^19^. Ca^2+^ controls essential cell functions, including proliferation, differentiation, secretion, contraction, metabolism, trafficking, and gene transcription and apoptosis. To investigate if the Ca^2+^ signaling was involved in up-regulation of ANKRD1 in HCV infected cells, Huh7.5 cells were treated with various doses of CaCl_2_ and then mRNA levels of ANKRD1 were analyzed by qRT-PCR. Figure 4A showed that mRNA levels of ANKRD1 were significantly increased by CaCl_2_. Consistently, protein levels of ANKRD1 were also gradually increased by CaCl_2_ in a dose dependent manner. To corroborate these results, Huh7.5 cells were either mock infected or infected with Jc1 for 6 days. The cells were treated with either intracellular Ca^2+^ chelator (BAPTA/AM) or extracellular Ca^2+^ chelator (EGTA). As expected, ANKRD1 expression was increased by HCV infection (Fig. 4B, lane 2). However, HCV-induced ANKRD1 expression levels were gradually decreased with increasing amounts of BAPTA-AM. Meanwhile, EGTA displayed no effect on ANKRD1 expression (Fig. 4B, lane 6), indicating that intracellular Ca^2+^ signaling was involved in up-regulation of ANKRD1 in HCV infected cells. Since ER is the main intracellular Ca^2+^ reservoir and alteration of Ca^2+^ homeostasis causes ER stress, we next investigated the effect of ER stress on ANKRD1 expression. Huh7.5 cells were either left untreated or treated with tunicamycin and then mRNA levels of ANKRD1 were analyzed by qRT-PCR. As shown in Fig. 4C (upper), mRNA levels of ANKRD1 were ~60 times increased by treatment with tunicamycin. Consistently, protein levels of ANKRD1 were remarkably increased by tunicamycin (Fig. 4C, lower). As expected, the expression of GRP78, a well-established ER stress marker, was prominently increased upon treatment with tunicamycin. Next, we assessed the involvement of ROS in up-regulation of ANKRD1 expression by HCV infection. As shown in Fig. 4D, H_2_O_2_ exerted no effect on ANKRD1 expression level (upper panel). Likewise, N-acetylcysteine (NAC), antioxidant, displayed no effect on ANKRD1 protein expressions in HCV infected cells. These data indicated that up-regulation of ANKRD1 expression was mediated through perturbation of intracellular calcium level and ER stress but not via ROS.

### ANKRD1 is required for HCV propagation

**Figure 5:**
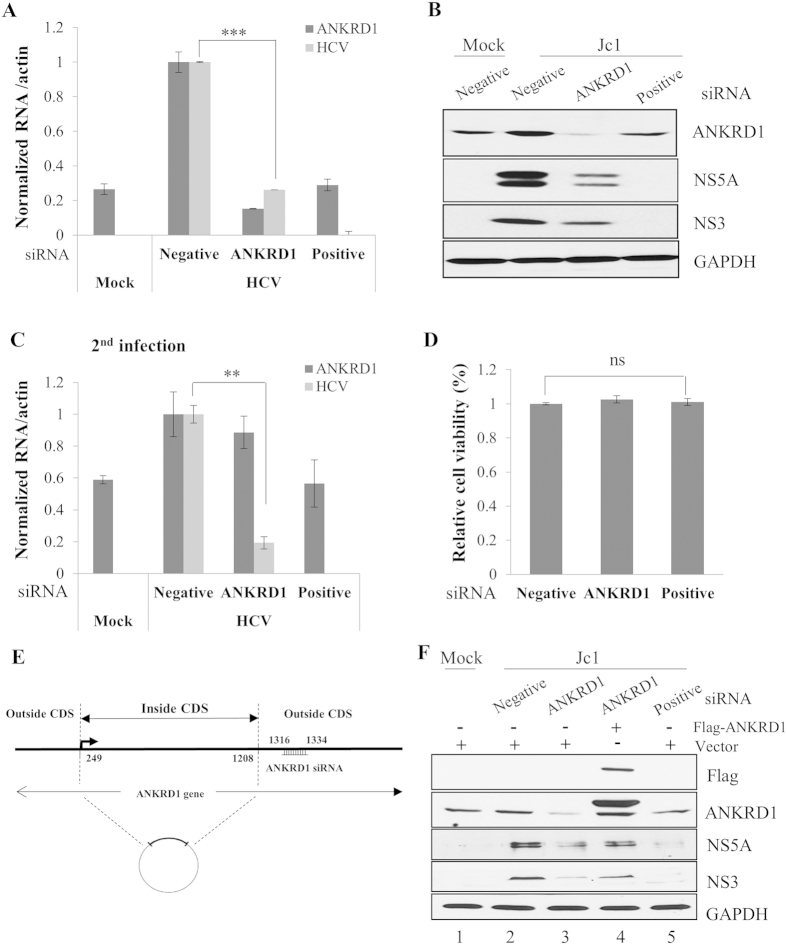
ANKRD1 is required for HCV propagation. (**A**) Huh7.5 cells were transfected with 20 nM of the indicated siRNAs for 48 h and then either mock infected or infected with Jc1 for 4 h. At 2 days postinfection, intracellular RNA levels were determined by qRT-PCR. Data represent averages from at least three independent experiments. The asterisks indicate significant differences (**P < 0.01; ***P < 0.001). (**B**) Huh7.5 cells were transfected with 20 nM of the indicated siRNAs for 48 h and then either mock infected or infected with Jc1 for 4 h. At 2 days postinfection, total cell lysates were immunoblotted with the indicated antibodies. Positive indicates HCV-specific siRNA targeting the 5′ nontranslated region (NTR) of Jc1. Negative depicts universal negative-control siRNA. (**C**) Naïve Huh7.5 cells were infected with Jc1 harvested from (**B**) for 4 h. At 48 h postinfection, intracellular HCV RNA levels were analyzed by qRT-PCR to determine infectivity. The asterisks indicate significant differences (**P < 0.01). (**D**) Huh7.5 cells were transfected with 20 nM of the indicated siRNAs. At 96 h after transfection, cell viability was determined by MTT assay. (**E**) Schematic diagram showing the siRNA target site outside of ANKRD1 coding sequence (CDS). (**F**) Huh7.5 cells were transfected with 20 nM of the indicated siRNAs for 24 h. The cells were further transfected with either vector or Flag-tagged ANKRD1 plasmid for 24 h. Cells were then either mock infected or infected with Jc1. At 48 h postinfection, total cell lysates were immunoblotted with the indicated antibodies.

To further investigate the functional involvement of ANKRD1 in HCV propagation, Huh7.5 cells transfected with siRNA targeting ANKRD1 or the indicated control siRNA constructs were infected with Jc1. At 48 h postinfection, both RNA and protein levels were determined. Silencing of ANKRD1 significantly impaired HCV RNA level (Fig. 5A). Consistently, knockdown of ANKRD1 suppressed HCV protein levels (Fig. 5B). To determine HCV infectivity, naïve Huh7.5 cells were infected with Jc1 harvested from Fig. 5A. As shown in Fig. 5C, HCV RNA level was significantly suppressed in ANKRD1 knockdown cells. Because transfection of siRNAs displayed no cytotoxicity (Fig. 5D), the silencing effect was specific to ANKRD1 in HCV-infected cells. In the present study, we used the siRNA targeting outside of coding sequence (CDS) region of ANKRD1 gene (Fig. 5E). To rule out the off-target effect of ANKRD1 siRNA, we performed recovery experiment using Flag-tagged ANKRD1 plasmid consisting of CDS. Huh7.5 cells were transfected with siRNA targeting ANKRD1 or the indicated control siRNA constructs. At 24 h after transfection, cells were further transfected with Flag-tagged ANKRD1 for 24 h and then infected with Jc1. At 48 h postinfection, protein levels were determined. As shown in Fig. 5F, exogenous expression of ANKRD1 rescued HCV protein expression (lane 4) as compared with vector transfected cells. Collectively, these data suggest that ANKRD1 is required for HCV propagation.

### ANKRD1 is not involved in the replication, translation, and virion production steps in the HCV life cycle

**Figure 6:**
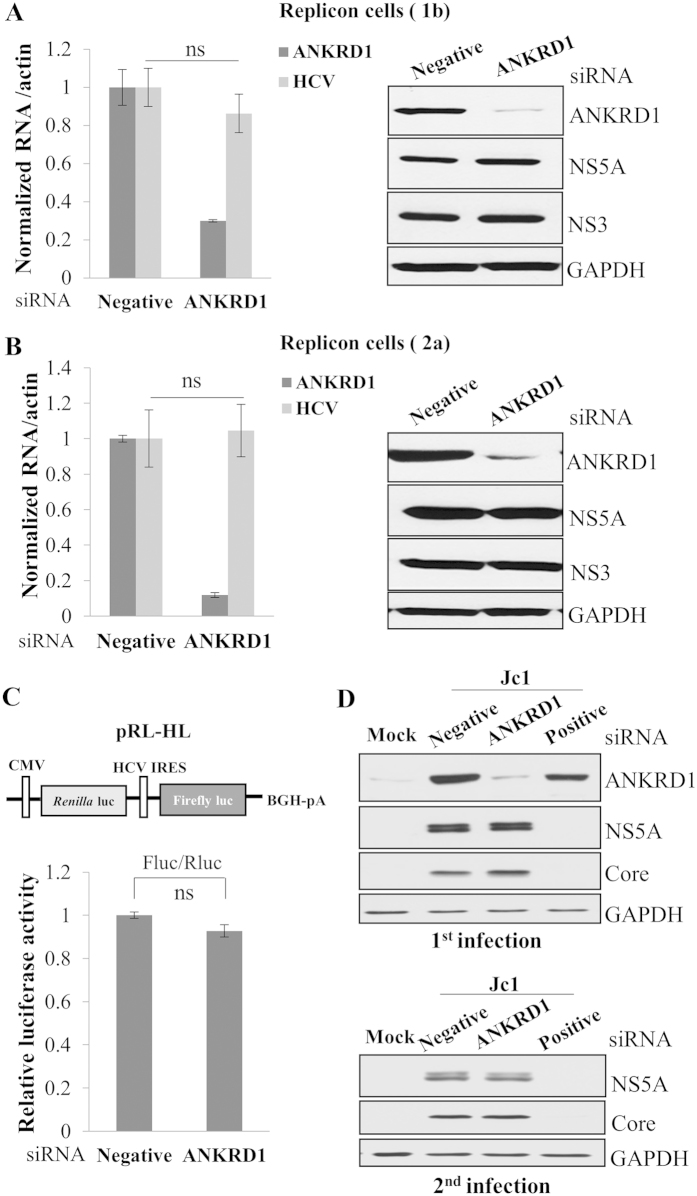
ANKRD1 is not involved in the replication step of the HCV life cycle. Cells harboring HCV subgenomic replicon derived from either genotype 1b (**A**) or genotype 2a (**B**) were transfected with 20 nM of the indicated siRNAs. At 72 h after transfection, both ANKRD1 RNA and HCV RNA levels were determined by qRT-PCR (left panels in **A**,**B**). Data represent averages from at least three independent experiments. Total cell lysates harvested at 72 h after transfection were immunoblotted with the indicated antibodies (right panels in **A**,**B**). (**C**) (Upper) Schematic diagram of pRL-HL plasmid. (Lower) Huh7.5 cells were transfected with the indicated siRNAs for 48 h and then further transfected with pRL-HL plasmid. At 48 h after transfection of the reporter plasmid, cells were harvested and luciferase activities were determined. ns, non-significant. (**D**) (Upper) Huh7.5 cells were either mock infected or infected with Jc1 for 48 h and then cells were transfected with the indicated siRNAs. At 48 h after transfection, total cell lysates were immunoblotted with the indicated antibodies. (Lower) Naïve Huh7.5 cells were infected with culture supernatant harvested from the above. At 48 h postinfection, total cell lysates were immunoblotted with the indicated antibodies. Positive indicates HCV-specific siRNA targeting the 5′ NTR of Jc1. Negative denotes universal negative-control siRNA.

To identify which step of the HCV life cycle required for ANKRD1, we first explored the possible involvement of ANKRD1 in HCV replication using subgenomic replicon cells derived from genotype 1b and 2a. As shown in Fig. 6A, silencing of ANKRD1 displayed no effect on HCV RNA levels in replicon cells derived from genotype 1b (left panel). Consistently, knockdown of ANKRD1 showed no effect on HCV protein expressions (Fig. 6A, right panel). We further confirmed that knockdown of ANKRD1 displayed no discernible effects on HCV RNA and protein expression levels in HCV subgenomic replicon cells derived from genotype 2a (Fig. 6B). These data indicate that ANKRD1 is not involved in replication step of the HCV life cycle. We next investigated whether ANKRD1 is involved in HCV internal ribosome entry site (IRES)-mediated translation. To address this question, Huh7.5 cells were transfected with the indicated siRNAs and then transfected with pRL-HL and β-galactosidase plasmid as we reported previously^20^, and then luciferase activity was determined. We demonstrated that silencing of ANKRD1 exerted no effects on HCV IRES-dependent translation (Fig. 6C). To further investigate if ANKRD1 was involved in other steps of the HCV life cycle, Huh7.5 cells were infected with Jc1 for 48 h and then transfected with either siRNA targeting ANKRD1 or the indicated control siRNA constructs. At 48 h post transfection, viral protein expressions were analyzed with the indicated antibodies. Figure 6D showed that knockdown of ANKRD1 displayed no effect on HCV protein levels in Jc1 infected cells (upper panel). To further analyze the possible involvement of ANKRD1 in virion production, naïve Huh7.5 cells were infected with Jc1 harvested from the supernatant of the 1^st^ infection. Figure 6D showed that HCV protein levels were not altered in cells re-infected with Jc1 from ANKRD1 knockdown cells (lower panel). All these data suggest that ANKRD1 was not involved in the replication, translation, and virion production steps in the HCV life cycle.

### ANKRD1 is a cellular factor required for HCV entry

**Figure 7:**
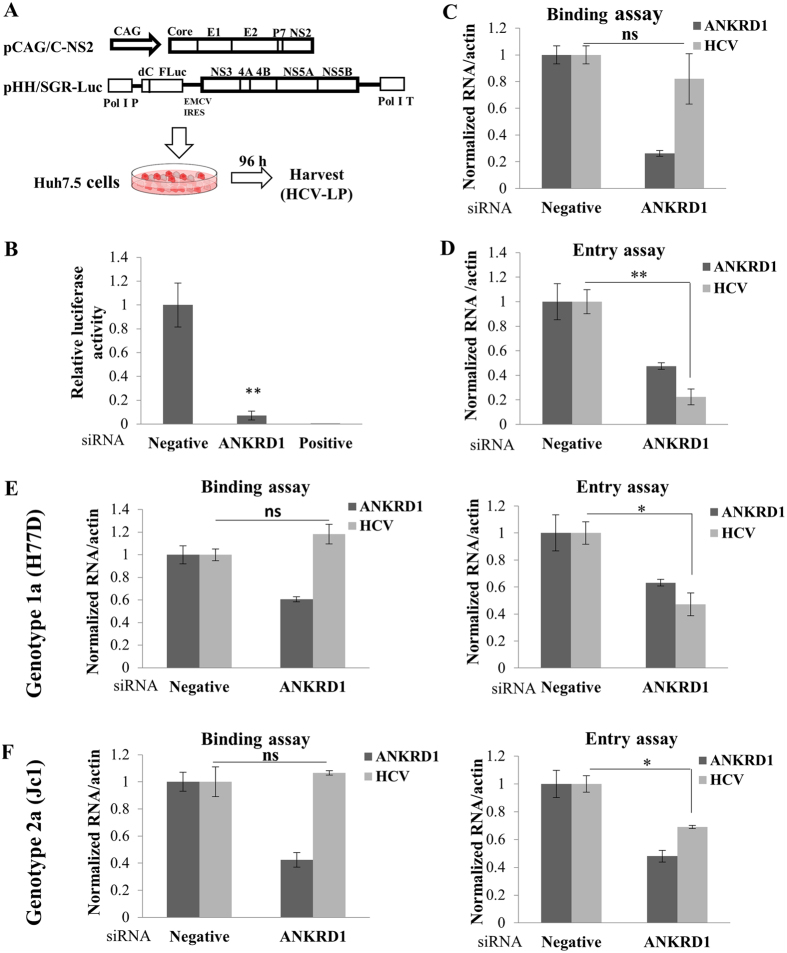
ANKRD1 is required in the entry step of the HCV life cycle. (**A**) Schematic diagram of the plasmids used for the production of HCV-LP. (**B**) Huh7.5 cells were transfected with the indicated siRNAs. At 48 h after transfection, cells were infected with HCV-LP. At 48 h postinfection, total cells were harvested and luciferase activities were determined for single cycle infection. Data represent averages from at least three independent experiments. The asterisks indicate significant differences (**P < 0.01) from the activity for the negative control. Positive indicates HCV-specific siRNA targeting the 5′ NTR of Jc1. Negative denotes universal negative-control siRNA. (**C**) Huh7.5 cells were transfected with the indicated siRNAs for 48 h. The cells were incubated with Jc1 at 4 °C for 2 h for binding. The cells were washed with PBS and then bound virions were determined by analyzing RNA levels by RT-PCR. (**D**) Huh7.5 cells were transfected with the indicated siRNAs for 48 h. The cells were incubated with Jc1 at 4 °C for 2 h. The cells were washed with PBS and then temperature was shifted to 37 °C for 4 h. The cells were trypsinized and washed twice with PBS to remove free virions. Internalized HCV virions were indirectly determined by analyzing RNA levels by RT-PCR. (**E**) Human primary hepatocytes were treated with H77D as described in C for binding assay and D for entry assay. RNA levels were determined by qRT-PCR. (**F**) Human primary hepatocytes were treated with Jc1 as described in C for binding assay and D for entry assay. Either bound HCV virions or internalized HCV virions were determined by qRT-PCR. Asterisks in all graphs show statistical significance: *P < 0.05; **P < 0.01. ns, non-significant.

Since ANKRD1 was not involved in replication and production steps of the HCV life cycle, we hypothesized that ANKRD1 might act in the entry step of the HCV life cycle. To explore this possibility, we performed viral entry assays using HCV like particle (HCV-LP) as described previously^21^. In this system, HCV-LP can undergo single cycle infection without producing virion. We first produced HCV-LP as depicted in Fig. 7A. Huh7.5 cells were transfected with siRNA targeting ANKRD1 and then infected with HCV-LP for 48 h. Figure 7B shows that HCV-LP luciferase activity was significantly reduced in ANKRD1 knockdown cells as compared to the negative control, indicating that ANKRD1 might be involved in infection steps of the HCV life cycle. To investigate if the ANKRD1 was required for the entry step, we performed viral infection assays using HCVpp. We observed that HCVpp infection was not perturbed in ANKRD1 knockdown cells (Supplementary Fig. 4). This result suggested that ANKRD1 might be involved in post binding step during HCV infection. To explore this possibility, we divided HCV infection steps into attachment/binding and entry/fusion steps. To analyze if ANKRD1 was required during attachment/binding step, Huh7.5 cells were transfected with either ANKRD1-specific siRNA or negative control siRNA for 48 h and then incubated with Jc1 inoculum for 2 h at 4 °C. After washing with PBS, bound virions were determined by HCV RNA levels. As shown in Fig. 7C, silencing of ANKRD1 displayed no effect on HCV binding to the host cells. Alternatively, to analyze if ANKRD1 was required during entry/fusion step, Huh7.5 cells transfected with ANKRD1-specific siRNA for 48 h were incubated with Jc1 for 2 h at 4 °C and then washed with PBS to remove unbound virions. Temperature was shifted to 37 °C for 4 h to allow virion to internalize the host cells. The cells were then trypsinized and non-internalized virions were washed away with PBS. Virion entry was determined by analyzing intracellular HCV RNA levels. Figure 7D demonstrated that HCV RNA level was significantly decreased in ANKRD1 knockdown cells, indicating that ANKRD1 acted at a post-binding step in entry. To further verify the effects of ANKRD1 on viral infection using other genotype of HCV, human primary hepatocytes transfected with ANKRD1-specific siRNA were treated with HCV genotype 1a^22^ as described above. As shown in Fig. 7E, knockdown of ANKRD1 specifically impaired entry step of the HCV infection from genotype 1a, further confirming that ANKRD1 was required at an entry step of the HCV life cycle. Using human primary hepatocyte culture system, we further demonstrated that ANKRD1 was required for HCV entry (Fig. 7F). Although we demonstrated that ANKRD1 was required for HCV entry in both 1a and 2a genotypes, whether this effect is pangenotypic has not been determined due to unavailability of infectious clones of all genotypes. Taken together, these data indicate that ANKRD1 is specifically involved in viral entry step of the HCV life cycle.

## Discussion

HCV coopts host cellular proteins for its own propagation. Thus the identification of host factors involved in HCV propagation may provide an alternative strategy to develop novel classes of host-targeted antivirals. By RNA-Seq analysis, we identified ANKRD1 which was highly expressed in HCVcc-infected cells. ANKRD1 is known to modulate transcriptional regulation and is involved in the process of both heart development and heart disease. In the fetal heart, ANKRD1/CARP negatively regulates cardiac gene expressions^9^,^11^. It has been previously reported that the basal ANKRD1 expression level is the highest in the heart muscle, whereas its expression level in the liver tissue is very low^23^. Since ANKRD1 expression is inducible by stress response, protein expression level of ANKRD1 could be increased by various stresses^10^. This may facilitate virus infection in the liver. Previous study shows that ANKRD1 expression in hepatoma cells is increased by exposing cells to the apoptogenetic drug Fenretinide^16^. Deregulation of ANKRD1 has also been implicated in various virus replications. It has been reported previously that ANKRD1 is involved in HIV replication by the interaction between gp120 and α_4_β_7_ on B cells^24^,^25^,^26^. Moreover, ANKRD1 is up-regulated in all three types of human papillomavirus in response to infection^27^. Here, we observed that protein expression of ANKRD1 was up-regulated by HCV infection, which is consistent with prior study showing that ANKRD1 is one of 50 most highly up-regulated genes during acute HCV infection^28^. We further showed that NS5A played a major role in augmentation of ANKRD gene expression. Indeed, NS5A interacted with ANKRD1 through domain II of NS5A and C-terminal half region of ANKRD1. NS5A is a multifunctional protein which modulates many signaling pathways in HCV infected cells^29^,^30^,^31^,^32^,^33^. Since ANKRD1 expression is also induced by IL-1 and TNF-alpha stimulation^10^, up-regulation of ANKRD1 in HCV infected cells may be mediated through protein interplay between NS5A and ANKRD1. However, additional studies are needed to better understand the implication of virus-host cell interaction in HCV propagation.

We assessed the functional role of ANKRD1 in HCV propagation. Knockdown of ANKRD1 impaired both HCV RNA and HCV protein levels. Silencing of ANKRD1 significantly decreased HCV infectivity without causing cellular toxicity. We therefore investigated which step of the HCV life cycle required for ANKRD1. We showed that ANKRD1 was not involved in replication, IRES-mediated translation, and virion production steps in the HCV life cycle. To further dissect the role of ANKRD1 in HCV life cycle, we employed HCV-LP assay. We observed that luciferase activity of HCV-LP was drastically decreased in ANKRD1 knockdown cells. Since ANKRD1 was not involved in RNA replication, translation, and virion production, this suggested that ANKRD1 might be involved in the entry step of the HCV life cycle. To verify whether ANKRD1 was involved in the viral entry, we performed HCVpp infection assay. However, HCVpp infection level was not altered in ANKRD1 knockdown cells. Since HCVpp assay does not completely capture all the entry steps of infectious HCV^34^, we divided the HCV infection stage into attachment/binding and entry/fusion steps, and analyzed precise involvement of ANKRD1 in HCV entry. Silencing of ANKRD1 displayed no effect on HCV binding. However, HCV entry was significantly impaired in ANKRD knockdown cells. We therefore provide the first evidence that ANKRD1 is the host factor required for a post binding step in HCV entry. HCV entry is a complex process that requires five cell surface molecules: CD81, scavenger receptor class B type I (SR-BI), claudin 1 (CLDN1), occludin (OCLN) and Niemann-Pick C1-like 1cholesterol absorption receptor (NPC1L1). Post binding step of HCV is the internalization via clathrin-mediated endocytosis^35^,^36^,^37^. Although we showed that ANKRD1 was involved in the entry step but not replication, translation or later steps of the HCV life cycle, the precise role of ANKRD1 in HCV entry needs further studies.

Using protein microarray assay, we recently reported that Pim kinase interacts with NS5A protein and regulates HCV entry^38^. We demonstrated that Pim kinase was specifically required at an early entry step of the HCV life cycle. In the present study, we performed transcriptome analyses and demonstrated that ANKRD1 was required for HCV entry. It is interesting that both Pim kinase and ANKRD1 are involved in HCV entry. Growing evidence shows that multiple host proteins are involved in HCV entry. We therefore hypothesize that HCV entry may be a complicated process involving the coordination of multiple cellular factors. We are uncertain that how Pim kinase and ANKRD1 are connected in HCV entry. Since HCV has evolved a number of strategies to facilitate viral infection on host cells, HCV may coopt various cellular proteins at different infection steps of the HCV life cycle for its own propagation.

We showed that ANKRD1 expression was up-regulated in both HCV replicon cells and HCV infected cells, and NS5A played a crucial role in ANKRD1 gene expression. We observed that both RNA and protein levels ANKRD1 were increased by CaCl_2_ in a dose dependent manner. We further showed that up-regulation of ANKRD1 expression in HCV infected cells was mediated through intracellular Ca^2+^ signaling. Since perturbation of Ca^2+^ signaling causes ER stress, we analyzed the effect of ER stress on ANKRD1 expression. Indeed, both RNA and protein levels ANKRD1 were increased by ER stress but not by ROS, indicating that HCV up-regulated ANKRD1 expression through Ca^2+^-mediated ER stress. Among HCV proteins, ANKRD1 expression was increased only by NS5A protein. Since NS5A induces oxidative stress-mediated Ca^2+^ homeostasis alterations in hepatocytes^17^,^39^, it is possible that Ca^2+^ and ER stress-mediated up-regulation of ANKRD1 may also play a role in the pathogenesis of liver diseases associated to HCV infection. Thus, it is likely that ANKRD1 is not only involved in the HCV entry but also contributes to HCV-associated pathogenesis. A growing body of evidence shows that certain host proteins exert multiple functions during the HCV life cycle. Recent study demonstrates that AP-2-associated protein kinase 1 and cyclin G-associated kinase regulates HCV entry as well as HCV assembly^40^. In addition, Fatty acid synthase is up-regulated during HCV infection and regulates both HCV entry and production^41^. Taken together, ANKRD1 is a novel key factor for HCV entry and thus it may represent a new target for antiviral drug development.

## Methods

### Plasmids and DNA transfection

Full-length ANKRD1 was amplified by primers (Supplementary Table 2) from cDNA synthesized from Huh7.5 cells by using cDNA synthesis kit (TOYOBO) according to manufacturer’s instructions. PCR products were inserted into the *Bgl*II*/Bam*HI sites of the plasmid pCMV10-3x Flag (Sigma Aldrich). ANKRD1 mutants were generated by PCR and subcloned into pCMV10-3x Flag vector. Plasmids expressing NS5A mutants, Myc-tagged HCV core, NS3, NS4B, NS5A, NS5B, and GST-NS5A were described elsewhere^42^. All DNA transfections were performed by using polyethyleneimine (Sigma-Aldrich) as we described previously^42^.

### ANKRD1 promoter assay

Human genomic DNA was isolated from Huh7 cells and ANKRD1 promoter was amplified by using PCR with the specific primers (Supplementary Table 2). The amplified PCR products were cloned into pGL3 vector (Promega Corp., Madison, WI) to generate luciferase reporter construct of ANKRD1 promoter. Luciferase assays were performed as we described previously^43^. Dual-luciferase reporter assay was described elsewhere^20^.

### Antibodies and reagents

ANKRD1/CARP, glyceraldehyde 3-phosphate dehydrogenase (GAPDH), and Myc antibodies were purchased from Santa Cruz; Flag antibody from Sigma-Aldrich; HCV core, NS3, and NS5A antibodies have been described elsewhere^42^. N-acetyl-cysteine (NAC), BAPTA/AM, and EGTA were purchased from Sigma-Aldrich.

### Cell Culture

All cell lines were grown in Dulbecco’s modified Eagle’s medium (DMEM) supplemented with 10% fetal bovine serum and 100 units/ml penicillin-streptomycin in 5% CO_2_ at 37 °C. Huh7 cells stably expressing NS5A or Huh7 cells harboring HCV subgenomic replicon were grown as reported previously^42^. Primary human hepatocytes were purchased from ScienCell Research Laboratories (CA, USA) and were cultured in hepatocyte media supplemented with 5% fetal bovine serum and 100 units/ml penicillin-streptomycin in 5% CO_2_ at 37 °C as we reported previously^38^.

### Immunoprecipitation

HEK293T cells were cotransfected with Flag-tagged ANKRD1 and Myc-tagged core, NS3, NS4B, NS5A, NS5B, respectively. Total amounts of DNA were adjusted by adding an empty vector. At 48 h after transfection, cells were harvested and an immunoprecipitation assay was performed using both anti-Myc and anti-Flag antibodies as we reported previously^42^.

### Immunoblot analysis

Equal amounts of proteins were subjected to SDS-PAGE and electrotransferred to a nitrocellulose membrane. The membrane was blocked in TBS/Tween (20 mM Tris-HCl [pH 7.6], 150 mM NaCl, and 0.25% Tween 20) containing 5% nonfat dry milk for 1 h at room temperature. The membrane was then incubated in TBS/Tween containing 1% nonfat dry milk overnight at 4 °C with the indicated antibodies. Following three washes in TBS/Tween, the membrane was incubated with either horseradish peroxidase-conjugated goat anti-rabbit antibody or goat anti-mouse antibody (Jackson ImmunoResearch Laboratories, West Grove, PA) for 1 h at room temperature. Proteins were detected using an ECL kit (Amersham Biosciences).

### RNA interference

siRNAs targeting ANKRD1 (sense, 5′-GUGAAGAUGUACCUAAUGA-3′; antisense, 5′-UACUUAGGUACAUCUUCAC-3′) and the universal negative control were purchased from Bioneer. siRNA targeting the 5′ nontranslated region (NTR) of Jc1 (5′-CCUCAAAGAAAAACCAAACUU-3′) was used as a positive control^42^. siRNA transfection was performed using a Lipofectamine RNAiMax reagent (Invitrogen, Carlsbad, CA) according to the manufacturer’s instructions.

### Quantification of ANKRD1 and HCV RNA

Total RNAs were isolated from HCVcc-infected cells, cell culture media, or replicon cells using RiboEx LS reagent (Geneall Biotechnology) and reverse transcribed using the ReverTra Ace kit (Toyobo). Quantitative real-time PCR (qRT-PCR) experiments were performed using an iQ5 multicolor Real-time PCR Detection system (Bio-Rad Laboratories, Hercules, CA) as we reported previously^44^.

### Single cycle HCV infection

Single cycle infectious HCV (HCVsc) was generated from a replicon *trans*-packaging system as previously described^21^. Briefly, Huh7.5 cells were cotransfected with pHH/SGR-Luc plasmid which carries a bicistronic HCV subgenomic replicon (NS3-5) firefly luciferase reporter with a Pol I promoter/terminator, and a HCV core-NS2 expression plasmid by using Lipofectamine 2000 reagent (Invitrogen, Carlsbad, CA) according to the manufacturer’s instructions. Culture medium containing HCV-like infectious particle (HCV-LP) was collected at 4 days after transfection. For single cycle HCV infection assay, Huh7.5 cells were treated with siRNA targeting either ANKRD1 or the indicated control siRNA before infection with HCVsc. At 48 h postinfection, cells were harvested and luciferase activity was determined.

### Binding and entry assays

Either Huh7.5 cells or primary human hepatocytes (0.6 × 10^5^ cells/well of 12-well plate) were transfected with either negative control siRNA or AKNRD1 siRNA for 48 h. For HCV binding assay, cells were incubated with either Jc1 at an MOI of 1 or H77D at an MOI of 0.2 at 4 °C for 2 h to allow virions for binding but not to internalize the target cells. After washing cells with PBS, bound virions were measured by RT-PCR. For HCV entry assay, cells were incubated with either Jc1 or H77D at 4 °C for 2 h, washed with PBS, and then temperature was shifted to 37 °C for 4 h to allow virions to internalize the cells. Cells were then trypsinized and washed twice with PBS to remove non-internalized virions^45^,^46^. HCV entry was indirectly determined by analyzing intracellular HCV RNA levels by RT-PCR.

### MTT assay

Huh7.5 cells were seeded at 4 × 10^4^ cells in a 24-well plate and transfected with the indicated siRNAs. At 4 days after transfection, 3-(4,5-dimethylthiazol-2-yl)-2,5-diphenyl-2H-tetrazolium bromide (MTT) reagent (Sigma) was added to the cells and incubated at 37 °C for 2 h. Cell viability was determined as we reported previously^42^,^43^.

### Statistical analysis

Data are presented as means ± standard deviations (SD). Student *t* test was used for statistical analysis of the data. The asterisks in the figures indicate significant differences (*P < 0.05; **P < 0.01; ***P < 0.001; ns, not significant).

## Additional Information

**How to cite this article**: Than, T. T. *et al.* Ankyrin Repeat Domain 1 is Up-regulated During Hepatitis C Virus Infection and Regulates Hepatitis C Virus Entry. *Sci. Rep.* **6**, 20819; doi: 10.1038/srep20819 (2016).

## Supplementary Material

Supplementary Information
