# Supplementary material for: Ankyrin Repeat Domain 1 is Up-regulated During Hepatitis C Virus Infection and Regulates Hepatitis C Virus Entry
Source: Sci Rep. 2016 Feb 10;6:20819. doi: 10.1038/srep20819 (PMC4748412; doi:10.1038/srep20819)

**Ankyrin Repeat Domain 1 is Up-regulated During Hepatitis C Virus Infection and Regulates Hepatitis C Virus Entry**

Thoa T. Than^1^, Giao V.Q. Tran^1^, Kidong Son^1^, Eun-Mee Park^1^, Seungtaek Kim^2^, Yun-Sook Lim^1*^, and Soon B. Hwang^1*^

*^1^National Research Laboratory of Hepatitis C Virus, Ilsong Institute of Life Science, Hallym University, Anyang, South Korea, ^2^Institute of Gastroenterology, Department of Internal Medicine, Yonsei University College of Medicine, Seoul, South Korea*

**Supplementary Table 1**. List of gene transcripts that were up-regulated in HCV-infected cells.

| **Gene** | **Accession NO.** | **Jc1** | **Mock** | **Jc1/Mock** |  | **Gene** | **Accession NO.** | **Jc1** | **Mock** | **Jc1/Mock** |
| --- | --- | --- | --- | --- | --- | --- | --- | --- | --- | --- |
| NCF2 | NM_000433 | 28.2 | 1.2 | 23 |  | RIOK3 | NM_003831 | 32.9 | 13.9 | 2.4 |
| MT1E | NM_175617 | 40.1 | 2.9 | 14.2 |  | TRIM31 | NM_007028 | 28.3 | 11.9 | 2.4 |
| MT1F | NM_005949 | 29.3 | 2.7 | 11 |  | CTSH | NM_004390 | 29.8 | 12.7 | 2.4 |
| MT2A | NM_005953 | 62.9 | 6.4 | 10.1 |  | BMP2 | NM_001200 | 52.9 | 22.5 | 2.4 |
| NPPB | NM_002521 | 88.7 | 9.6 | 9.3 |  | TUBB3 | NM_006086 | 34.6 | 14.8 | 2.4 |
| STC2 | NM_003714 | 26.6 | 3.2 | 8.2 |  | JUN | NM_002228 | 28.5 | 12.2 | 2.3 |
| SERPINE2 | NM_006216 | 28.9 | 6.8 | 4.3 |  | TNFRSF21 | NM_014452 | 42 | 18.2 | 2.3 |
| RCN1 | NM_002901 | 33.7 | 4.5 | 7.5 |  | DAB2 | NM_001343 | 30.1 | 12.8 | 2.3 |
| AREG | NM_001657 | 13.1 | 1.9 | 6.9 |  | DST | NM_015548 | 36.4 | 15.6 | 2.3 |
| TAGLN | NM_003186 | 50.6 | 7.9 | 6.3 |  | GTPBP2 | NM_019096 | 33.8 | 14.4 | 2.3 |
| ANKRD1 | NM_014391 | 101 | 16.2 | 6.2 |  | KLF6 | NM_001300 | 29.1 | 12.5 | 2.3 |
| HABP2 | NM_004132 | 29.9 | 5.2 | 5.7 |  | FBLIM1 | NM_017556 | 20.3 | 8.7 | 2.3 |
| IL18 | NM_001562 | 27.5 | 5.2 | 5.4 |  | PROM1 | NM_006017 | 55.9 | 24.3 | 2.3 |
| IL8 | NM_000584 | 109.6 | 20.3 | 5.3 |  | CTH | NM_001902 | 22.3 | 9.5 | 2.3 |
| CYP1A1 | NM_000499 | 77 | 15.9 | 5.3 |  | RHOC | NM_175744 | 149.2 | 64.3 | 2.3 |
| PRSS23 | NM_007173 | 28 | 5.4 | 5.2 |  | PKIB | NM_032471 | 15.4 | 6.7 | 2.3 |
| INHBE | NM_031479 | 22 | 4.5 | 4.9 |  | FAM65A | NM_024519 | 28.6 | 12.4 | 2.3 |
| FSTL1 | NM_007085 | 24.6 | 5.5 | 4.7 |  | HEG1 | NM_020733 | 30.3 | 13.2 | 2.3 |
| ANXA1 | NM_000700 | 99.8 | 21.5 | 4.6 |  | SEMA6A | NM_020796 | 22.7 | 9.8 | 2.3 |
| CALD1 | NM_033138 | 28.8 | 6.8 | 4.3 |  | RRAGD | NM_021244 | 31.8 | 14 | 2.3 |
| MT1X | NM_005952 | 39.3 | 9.2 | 4.3 |  | SELM | NM_080430 | 36.4 | 16.4 | 2.3 |
| ATF3 | NM_001674 | 37.8 | 9.1 | 4.1 |  | TUBB2B | NM_178012 | 49.9 | 21.6 | 2.3 |
| HDAC9 | NM_058176 | 27.9 | 6.9 | 4.1 |  | CDH17 | NM_004063 | 66.3 | 29.4 | 2.3 |
| IL32 | NM_004221 | 40.9 | 12.4 | 3.4 |  | CLIP1 | NM_002956 | 22.7 | 10.1 | 2.3 |
| HSPB8 | NM_014365 | 29 | 7.3 | 4 |  | ABCB1 | NM_000927 | 42.2 | 18.8 | 2.3 |
| DUSP1 | NM_004417 | 49.3 | 12.9 | 3.8 |  | ABCC3 | NM_003786 | 26.3 | 11.7 | 2.3 |
| GADD45B | NM_015675 | 20.8 | 5.7 | 3.6 |  | PVR | NM_006505 | 45.3 | 20.2 | 2.2 |
| UCA1 | NR_015379 | 39.1 | 11.3 | 3.5 |  | STBD1 | NM_003943 | 49.9 | 22.3 | 2.2 |
| THBS1 | NM_003246 | 68.7 | 21.2 | 3.3 |  | PTGR1 | NM_012212 | 89.3 | 42.3 | 2.1 |
| MRAS | NM_012219 | 29.2 | 9 | 3.2 |  | TPM1 | NM_001018005 | 21.7 | 9.8 | 2.2 |
| NRCAM | NM_005010 | 34.7 | 11.4 | 3.1 |  | HYI | NM_031207 | 29.9 | 13.5 | 2.2 |
| GDF15 | NM_004864 | 31.1 | 10.3 | 3 |  | SLC29A3 | NM_018344 | 18.7 | 8.4 | 2.2 |
| PAPSS2 | NM_004670 | 42.1 | 14.1 | 3 |  | PLA2G16 | NM_007069 | 118 | 55.1 | 2.1 |
| ANXA3 | NM_005139 | 109.5 | 37.2 | 2.9 |  | ELF3 | NM_004433 | 47.4 | 21.4 | 2.2 |
| SLC1A5 | NM_005628 | 84.9 | 30.6 | 2.8 |  | PLK2 | NM_006622 | 34.6 | 15.6 | 2.2 |
| CAV2 | NM_001233 | 29.2 | 10.1 | 2.9 |  | ENAH | NM_018212 | 225.7 | 102.3 | 2.2 |
| UGP2 | NM_006759 | 29.4 | 10.4 | 2.8 |  | AHR | NM_001621 | 70.5 | 32.2 | 2.2 |
| LGALS8 | NM_006499 | 29.7 | 10.9 | 2.7 |  | CRIM1 | NM_016441 | 33.8 | 15.3 | 2.2 |
| CYR61 | NM_001554 | 65.8 | 23.8 | 2.8 |  | ITGAV | NM_002210 | 15.9 | 7.2 | 2.2 |
| RAB32 | NM_006834 | 52.1 | 20.6 | 2.7 |  | ETS2 | NM_005239 | 39.8 | 18.1 | 2.2 |
| SLC3A2 | NM_002394 | 271.5 | 101 | 2.7 |  | TNFRSF12A | NM_016639 | 33.1 | 15.2 | 2.2 |
| PHLDA1 | NM_007350 | 26.4 | 10.2 | 2.7 |  | TRIB1 | NM_025195 | 17.9 | 8.2 | 2.2 |
| SPINK1 | NM_003122 | 711.3 | 262.6 | 2.7 |  | MICAL3 | NM_015241 | 21.3 | 9.8 | 2.2 |
| KLF10 | NM_005655 | 18.6 | 6.9 | 2.7 |  | SLC12A2 | NM_001046 | 116.1 | 54 | 2.2 |
| OPTN | NM_021980 | 70.4 | 26.5 | 2.7 |  | CTSL1 | NM_001912 | 79.4 | 37.1 | 2.2 |
| UACA | NM_018003 | 38.2 | 14.8 | 2.6 |  | PALLD | NM_016081 | 48.4 | 22.7 | 2.1 |
| CDH2 | NM_001792 | 53.9 | 20.6 | 2.6 |  | CAV1 | NM_001753 | 25.6 | 11.9 | 2.2 |
| VIM | NM_003380 | 1229.4 | 475.8 | 2.6 |  | IRAK2 | NM_001570 | 17.5 | 8.1 | 2.2 |
| ABLIM1 | NM_002313 | 35.6 | 14.4 | 2.6 |  | GBA | NM_000157 | 68.1 | 32.1 | 2.1 |
| MOSPD1 | NM_019556 | 25.5 | 10.3 | 2.6 |  | DPP4 | NM_001935 | 108.8 | 50.6 | 2.1 |
| FZD5 | NM_003468 | 55.3 | 21.4 | 2.6 |  | SH3BP5 | NM_004844 | 24 | 11.2 | 2.1 |
| STX3 | NM_004177 | 117.8 | 46.4 | 2.6 |  | G6PD | NM_000402 | 53.7 | 25 | 2.1 |
| WARS | NM_004184 | 28.5 | 11.1 | 2.6 |  | AFF4 | NM_014423 | 22.9 | 10.7 | 2.1 |
| TRIB3 | NM_021158 | 44.2 | 17.4 | 2.6 |  | MYL9 | NM_006097 | 28.4 | 13.4 | 2.1 |
| OSGIN1 | NM_013370 | 29.2 | 11.4 | 2.6 |  | MMP14 | NM_004995 | 30.8 | 14.5 | 2.1 |
| CDKN2B | NM_004936 | 17.2 | 6.7 | 2.6 |  | MTHFD2 | NM_006636 | 116.8 | 55 | 2.1 |
| LOXL4 | NM_032211 | 32.9 | 12.9 | 2.6 |  | KLHL5 | NM_015990 | 42.1 | 20.3 | 2.1 |
| CPT1A | NM_001876 | 29.8 | 11.7 | 2.5 |  | HEXB | NM_000521 | 69.7 | 33.5 | 2.1 |
| PPP1R15A | NM_014330 | 37.4 | 15.2 | 2.5 |  | MAF | NM_001031804 | 5.2 | 2.5 | 2.1 |
| SEL1L3 | NM_015187 | 65.3 | 25.9 | 2.5 |  | STAT3 | NM_003150 | 32.7 | 15.5 | 2.1 |
| TIPARP | NM_015508 | 26.1 | 10.3 | 2.5 |  | EHD4 | NM_139265 | 36.5 | 17.4 | 2.1 |
| SLC44A3 | NM_001114106 | 44.4 | 18.2 | 2.5 |  | OTUD7B | NM_020205 | 33.5 | 16 | 2.1 |
| PPIC | NM_000943 | 29.2 | 11.6 | 2.5 |  | BICC1 | NM_001080512 | 27.6 | 13.2 | 2.1 |
| NOSTRIN | NM_052946 | 39 | 15.9 | 2.5 |  | S100P | NM_005980 | 38 | 18.2 | 2.1 |
| LMCD1 | NM_014583 | 18.5 | 7.5 | 2.5 |  | MAP1LC3B | NM_022818 | 84.1 | 40.6 | 2.1 |
| SKAP2 | NM_003930 | 24.1 | 9.7 | 2.5 |  | HABP4 | NM_014282 | 18.1 | 8.8 | 2.1 |
| PCK2 | NM_004563 | 38.4 | 16.5 | 2.4 |  | TMCO3 | NM_017905 | 31.7 | 15.4 | 2.1 |
| MAP1B | NM_005909 | 31.9 | 13.4 | 2.4 |  | IQGAP1 | NM_003870 | 36.7 | 17.9 | 2.1 |
| FILIP1L | NM_182909 | 34.4 | 14.3 | 2.4 |  | F3 | NM_001993 | 26.7 | 13.1 | 2 |
| OCLN | NM_002538 | 18.5 | 7.8 | 2.4 |  | SLC1A4 | NM_003038 | 35.4 | 17.3 | 2 |
| WWC1 | NM_001161661 | 34.1 | 14.5 | 2.4 |  | BCAR1 | NM_001170714 | 22.2 | 11 | 2 |
| ACSL1 | NM_001995 | 28.7 | 12.2 | 2.4 |  | CEBPD | NM_005195 | 12.2 | 6 | 2 |
| ASNS | NM_001673 | 169.4 | 72.6 | 2.3 |  |  |  |  |  |  |

**Supplementary Table 2**. List of primers used in this study.

| **Primer** | **Primer sequence** | **Enzyme site** | **Purpose** |
| --- | --- | --- | --- |
| ANKRD1-F | CGAGATCTGATGATGGTACTGAAAGTAGAG | *Bgl*II | Cloning of human ANKRD1 into p3XFLAG-CMV-10 vector |
| ANKRD1-R | ATGGATCCGAGTCTGTCGTTT GCCTCAG | *Bam*HI |
| ANKRD1-F-domain | GCAGATCTGATGGAGTATAAACGGACA | *Bgl*II | Subcloning of human ANKRD1 truncated regions into p3XFLAG-CMV-10 vector |
| ANKRD1-R-domain | ACGGATCCTCAATCACAAACATCTGG | *Bam*HI |
| ANKRD1-F | ATGGTACCTTTGAGGTGATTGAAACAGTAAGGAGCCATTTAATCAG | *Kpn*I | Cloning of human ANKRD1 promoter into basic pGL3 vector |
| ANKRD1-R | GCTCGAGATGGACAAGCTACCCCTGCTGAATATGTGAATCAGGAAG | *Xho*I |
| ANKRD1-F | GCCTACGTTTCTGAAGGCTG |  | Quantitative real-time PCR |
| ANKRD1-R | GTGGATTCAAGCATATCACGGAA |  |
| 5'NTR-F (Jc1) | TGAGTGTCGTACAGCCTCCA |  | Quantitative real-time PCR |
| 5'NTR-R (Jc1) | ACGCTACTCGGCTAGCAGTC |  |
| 5'NTR-F (H77D) | TCTGCGGAACCGGTGAGTA |  | Quantitative real-time PCR |
| 5'NTR-R (H77D) | TCAGGCAGTACCACAAGGC |  |
| qActin-F | TGACAGCAGTCGGTTGGAGCG |  | Quantitative real-time PCR |
| qActin-R | GACTTCCTGTAACAACGCATCTCATA |  |

**Supplementary Methods**

***In vitro* pulldown assay.** Glutathione S-transferase (GST)-NS5A fusion protein was expressed in *E. coli* BL21 (DE) (Novagen) and purified with glutathione-Sepharose 4B beads. Total cell lysates containing Flag-tagged ANKRD1 were incubated with either GST or GST-NS5A protein for 1 h at 4°C. Bound protein was detected by immunoblot analysis using anti-Flag monoclonal antibodies.

**Transcriptome analysis.** Huh7.5 cells infected with Jc1 were cultured for 6 days. Cells were harvested and then total RNAs were extracted. One microgram of RNA was used to construct cDNA libraries using the TruSeq RNA library kit. The procedure was followed by polyA-selected RNA extraction, RNA fragmentation, random hexamer primed reverse transcription, and 100 nt paired-end sequencing by Illumina HiSeq2000. The libraries were quantified by qRT-PCR according to the Quantification Protocol Guide and qualified using an Agilent Technologies 2100 Bioanalyzer. RNA-seq and data analysis has been done by Macrogen (Korea).

**HCV** **pseudoparticle infection assay.** HCV pseudoparticles (HCVpp) with E1 and E2 glycoproteins derived from genotype 1a (H77) or genotype 2a (JFH-1) were generated as previously described^1,2^. Vesicular stomatitis virus pseudoparticle (VSVpp) were generated and used as a control.

**Supplementary References**

1. Bartosch, B., Dubuisson, J. & Cosset, F. L. Infectious hepatitis C virus pseudo-particles containing functional E1-E2 envelope protein complexes. *J. Exp. Med.* **197,** 633–642 (2003).

2. Matsumura, T. *et al*. 25-Hydroxyvitamin D3 suppresses hepatitis C virus production. *Hepatology* **56,** 1231–1239 (2012).

**Supplementary Figure Legends**

**Figure S1 Candidate genes that were highly differentially expressed in HCVcc-infected cells.** Huh7.5 cells were either mock infected or infected with Jc1. At day 6 postinfection, intracellular RNAs were analyzed by qRT-PCR. Data represent the relative RNA level of 30 genes that were highly differentially expressed in Jc1 infected cells as compared with the mock infected cells. Data are arranged in descending order.

**Figure S2** Huh7.5 cells were transfected with the indicated siRNAs for 2 days. Cells were infected with Jc1 for 4 h and harvested at 2 days postinfection. Total RNAs were extracted and RNA levels were measured by qRT-PCR.

**Figure S3** **ANKRD1 interacts with NS5A *in vitro.*** HEK293T cells were transfected with Flag-tagged ANKRD1. Total cell lysates harvested at 24 h after transfection were incubated with either GST or GST-NS5A protein. Protein complexes were precipitated with glutathione beads for 1 h at 4°C. Bound protein was detected by immunoblot analysis using anti-Flag monoclonal antibodies. Arrow indicates GST-NS5A and arrowhead denotes GST.

**Figure S4** **Silencing of ANKRD1 displayed no effect on HCVpp infection.** Huh7.5 cells were transfected with 20 nM of the indicated siRNAs for 48 h. Cells were then infected with either VSVpp or HCVpp and then viral entry was determined by luciferase activity at 48 h postinfection.

Supplementary Figure 1


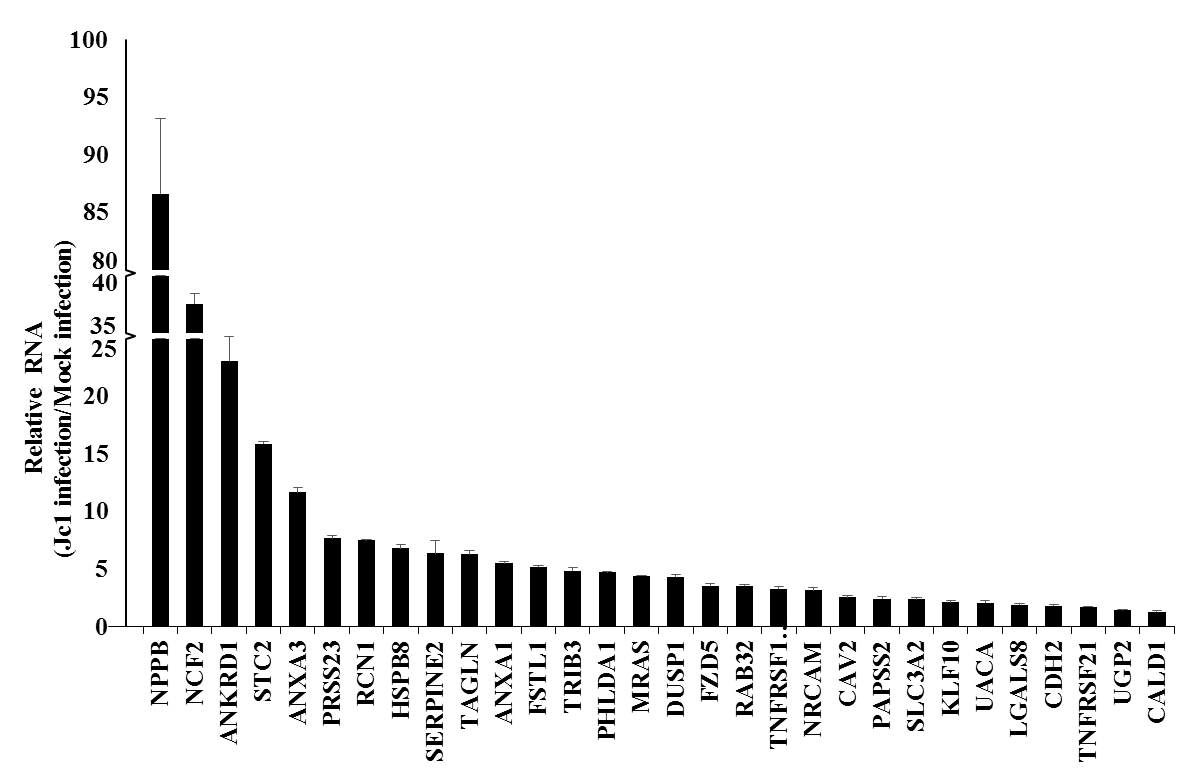


Supplementary Figure 2


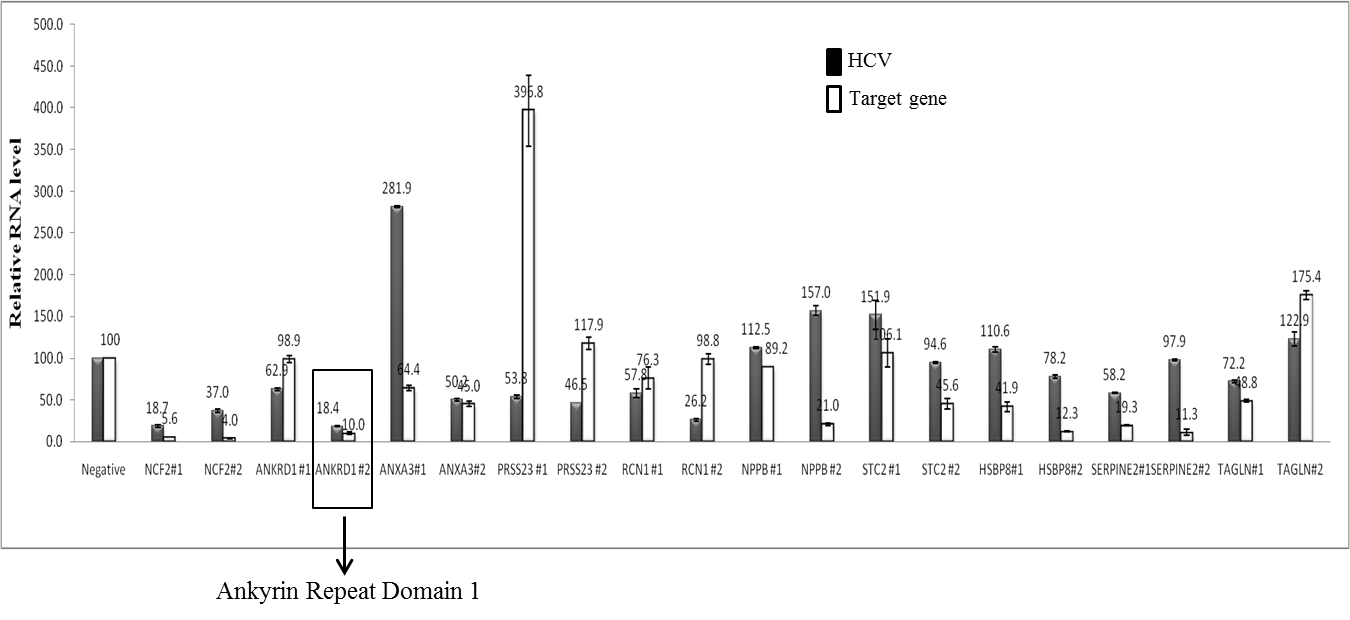


Supplementary Figure 3


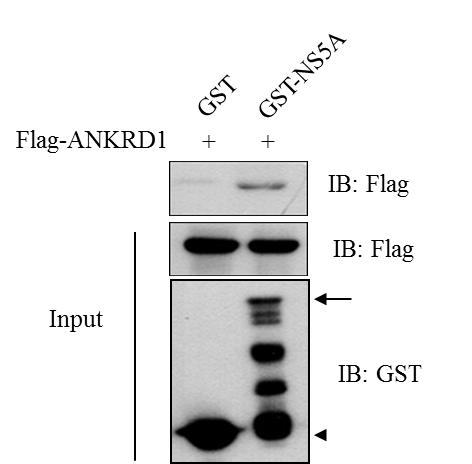


Supplementary Figure 4


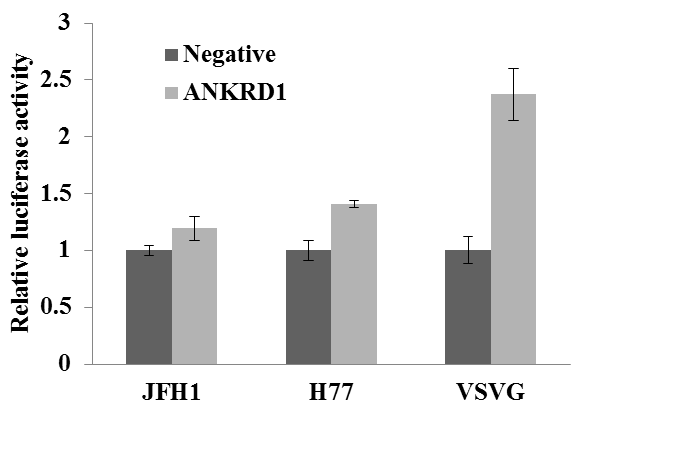

Supplement: Supplementary Information [file srep20819-s1.doc]
